# A comparison of the shaping ability of three nickel-titanium rotary instruments: a micro-computed tomography study via a contrast radiopaque technique in vitro

**DOI:** 10.1186/s12903-016-0326-5

**Published:** 2017-01-09

**Authors:** Zhao Wei, Zhi Cui, Ping Yan, Han Jiang

**Affiliations:** 1 Department of Endodontics, School and Hospital of Stomatology, Wuhan University, 237 Luoyu Road, Wuhan, 430079 China; 2 The State Key Laboratory Breeding Base of Basic Science of Stomatology (Hubei-MOST) and Key Laboratory of Oral Biomedicine Ministry of Education, School and Hospital of Stomatology, Wuhan University, Luoyu Road 237, Wuhan, 430079 China

**Keywords:** Meglucamine diatrizoate, Micro-computed tomography, Nickel-titanium rotary instruments, Shaping ability, Simulated root canals

## Abstract

**Background:**

Micro-CT (μCT) studies that combine simulated canals with meglucamine diatrizoate to evaluate the shaping ability of nickel-titanium (NiTi) rotary instruments are lacking in the literature. The purpose of this study was to evaluate the shaping ability of three new different nickel-titanium rotary instruments in simulated root canals using μCT.

**Methods:**

Thirty simulated root canals with a curvature of 60° were randomly allocated into the following 3 groups (*n* = 10): Group 1, ProTaper Universal (PTU) rotary system; Group 2, Reciproc single-file system; and Group 3, K3XF rotary system. Pre- and post-instrumented images of simulated canals were scanned with μCT via a radiopaque contrast technique to build a 3-dimensional (3D) model. Canal transportation, volumetric change and centring ability were evaluated in each group. Instrument failure and preparation time were also recorded. The Kruskal-Wallis test was used for statistical analysis and the significance level was set at *p* = 0.05.

**Results:**

Reciproc produced greater volume change in the apical part of the canals compared with PTU and K3XF (*p* < 0.05). K3XF exhibited less transportation and better centring ability at the 2- and 3-mm levels from the apical foramen compared with PTU and Reciproc (*p* < 0.05). There were no significant differences in the centring ratio and transportation between PTU and Reciproc. Preparation time was significantly shorter in the Reciproc group (*p* < 0.05).

**Conclusions:**

Under the conditions of our study, all of the canals were 3D reconstructed successfully via the radiopaque contrast technique. Reciproc showed enhanced apical volume changes and K3XF exhibited better centring ability when compared with other groups.

**Electronic supplementary material:**

The online version of this article (doi:10.1186/s12903-016-0326-5) contains supplementary material, which is available to authorized users.

## Background

Successful root canal therapy relies on the effective shaping and debridement of the root canal system without damaging the original configuration [1]. Preparation by using manual or rotary instrumentation techniques in a canal may lead to procedural errors, such as transportation, ledging, zipping and elbowing, however, various studies showed that NiTi rotary instruments maintained original canal shape better than stainless steel files [2, 3]. The elasticity and resistance to torsional fracture of NiTi rotary instruments make it possible to prepare curved canals in a relatively effective and safe manner [4, 5]. Over the decades, various NiTi rotary systems have been introduced to the market and each of them has a different design for its tip, taper, pitch, and rake and helical angles. In attempts to reduce the incidence of fracture and enhance the shaping ability, recent research has focused on new methods or techniques to improve the mechanical properties of NiTi instruments, including the use of R-phase or M-wire alloys and modifications in the design of the cross-section, reciprocating or rotary working motion.

The ProTaper Universal (PTU) rotary system (Dentsply Maillefer Ballaigues, Switzerland) was launched several years ago. According to the manufacturer, the convex cross-sectional design with a shallow U-shaped groove in each convex triangular edge seeks to improve the flexibility of instruments and reduce apical transportation. The cross section of finishing files F3, F4, and F5 are designed as concave shape for increasing the flexibility. Reciproc (VDW, Munich, Germany) is made of M-wire alloy that presents a higher fatigue resistance with a reduced risk for instrument fracture. With an S-shaped cross-section design and non-cutting tip, this single file works in a reciprocating motion. It was suggested that the use of reciprocating techniques could reduce the incidence of fracture. The K3XF rotary system (SybronEndo, Orange, CA) has been available with an updated alloy design compared with the K3 system. The mechanical properties are modified by the special heat treatment (R-phase technology) on the alloy during the manufacturing process. This system was reported to have a higher fracture resistance than traditional NiTi rotary files [6]. The differences in the metallurgy and design of these modified NiTi instruments may produce different shaping abilities, and clinicians are often confused about which is better for an individual case to produce more centred preparations and minimize apical root canal transportation.

Various techniques involving simulated canals or extracted teeth have been used to evaluate the shaping ability of endodontic instruments. Traditionally, the evaluation of the shaping ability of endodontic instruments are usually performed using two-dimensional (2D) techniques, which rely on precisely fixed devices for taking radiographs/images and using software for calculation [7–9]. These 2D techniques are inexpensive and allow for the simultaneous comparison of the root canal morphology before and after preparation; however, the results of these studies are inaccurate and volumetric information was not disclosed.

Micro-computed tomography (μCT) is a non-invasive method for the three-dimensional evaluation of the shaping ability and the basic geometric parameters of the tooth’s internal morphology [8, 10]. Data generated by μCT scanning can be analysed quantitatively and qualitatively with software, which can provide accurate information on volumetric changes, surface area, canal transportation, and canal diameter. Ounsi et al. reported that μCT is more sensitive to the changes in the canal space compared with 2D techniques [11]. μCT scanning is always used in extracted human teeth to evaluate the shaping ability of instruments. Notably, there is evidence to show the correlation between the complexity of root canal anatomy and the quality of root canal instrumentation [12]_._ Although it is often ignored characterizing, the heterogeneity of data drawn from extracted teeth with anatomic variations is very important for evaluating the shaping ability of endodontic instruments. This problem could be addressed by using standardized canals in resin blocks with the same length, taper, diameter and radius of curvature for data analysis. The reliability of the simulated canal as an ideal model for the analysis of instrumentation techniques has been validated [13]. However, due to the high radiolucency of resin blocks and the difficulty of 3D image reconstruction of simulated canals, few studies have been conducted to compare the shaping ability of endodontic instruments using μCT reconstruction techniques with standardized simulated canals.

Meglucamine diatrizoate is a water-soluble radiocontrast medium widely used for contrast-enhanced CT examination in patients for radiographic diagnosis and treatment [14]. It was verified that the combination of spiral CT and meglucamine diatrizoate could produce high-quality images for data analysis of 3D-reconstructed moulds [15].

Until now, μCT studies that combine simulated canals with meglucamine diatrizoate are lacking in the literature. In addition, no study to date has directly compared PTU, Reciproc and K3XF with regard to their shaping ability. The aim of our study was to comparatively analyse the canal shaping ability of PTU, Reciproc and K3XF by μCT in simulated canals via the meglucamine diatrizoate radiocontrast technique. The null hypothesis was that there would be no difference among the 3 rotary file systems regarding the analyzed parameters.

## Methods

### Simulated canals

Thirty simulated root canals with a single curvature in clear resin blocks (Endo Training Bloc; Dentsply Maillefer, Ballaigues, Switzerland) were used for this study. All canals were produced with a 0.15 mm apical foramen, 0.02 taper, 16.5 mm canal length, a 60°curvature (4.5 mm radius) and a 10-mm straight section before the curve [16]. Subsequently, the blocks were randomly assigned to 3 groups (n = 10): PTU, Reciproc and K3XF. We defined the direction of the apical foramen as distal.

### Instrumentation of simulated canals

The instrumentation of all blocks was carried out by one experienced operator. Each instrument was only used to shape one canal. The patency of the root canals was confirmed with a stainless steel #10 K-file (Dentsply Maillefer). And a glide path was established in each canal using #13, #16 and #19 PathFiles (Dentsply Maillefer) at 300 rpm to the working length (WL), which was determined by inserting #10 K-file to the canal terminus and subtracting 0.5 mm from the measurement. The instrumentation sequences were then carried out as follows:

### Group 1

Root canals were prepared with the PTU system. According to manufacturer’s guidelines, all canals assigned to this group were shaped from S1 to F2 using the crown-down technique in full clockwise rotation by using X-Smart Plus endodontic motor (Dentsply Maillefer) with a 16:1 reduction handpiece. The torque and rotational speed were adjusted according to the manufacturer’s recommendations. The F2 was used to standardize the diameter at the end-point of preparation to 0.25 mm, and the canal taper was 0.08.

### Group 2

Reciproc files (R25, #25/0.08) were used in this group. Files were operated in a programmed slow in-and-out pecking, reciprocating motion according to the manufacturer’s instructions. The flutes of the instrument were cleaned after 3 movements and then the canal was irrigated with sterile water. X-Smart Plus endodontic motor in the “RECIPROC” mode was used in this procedure.

### Group 3

K3XF were used in this group. Ten blocks were prepared with a 16:1 reduction handpiece powered by the X-Smart Plus endodontic motor, set at 350 rpm and 3 Ncm torque. The following crown-down sequence was used: 25/10: 11 mm, 25/08: 14 mm, and 25/06: 16 mm (WL).

Following each file, patency was verified with #10 K-file and canal was irrigation with sterile water using a 27-gauge side-vented needle. The time for instrumentation was recorded which included only the time for active instrumentation. The number of permanently deformed instruments and fractured instruments during these procedures were also recorded.

### Micro-CT (μCT) scanning and measurements

Pre- and post-instrumented images were scanned by μCT (μCT-50; Scanco Medical, Bassersdorf, Switzerland) with a resolution of 30 μm. Before scanning, all of the canals were brimming with 60% meglucamine diatrizoate injection. The parameters for the μCT scanner were 90 kVp and 88 μA. The cross-sectional images were segmented, visualized, registered, and quantified using analysis software (VGStudio Max version 2.2.0; Volume Graphics, Heidelberg, Germany). We selected the region extending from the constriction of the orifice to the apex as the area of interest. According to the number of cross-sectional slices, the artificial canals were equally divided into three parts, namely the coronal-, mid- and apical thirds of the canals.

### Measurement of canal volume

The volume change between pre- and post- instrumentation of the three parts of each canal were computed separately using VGStudio Max 2. 2. 0.

### Measurement of canal transportation

To evaluate the degree of canal transportation in the mesio-distal direction, these cross-sections were determined using perpendicular lines made by the canal axes at 1, 2, 3, 5, and 7 mm. Images of the cross-sections pre- and post- instrumentation were superimposed and recorded to determine the canal transportation and centring ability. The absolute value was calculated for each cross-section using the following formula [17]:

$$ \left|\left(\mathrm{X}1\hbox{--} \mathrm{X}2\right)-\left(\mathrm{Y}1\hbox{--} \mathrm{Y}2\right)\right| $$

**Fig. 1:**
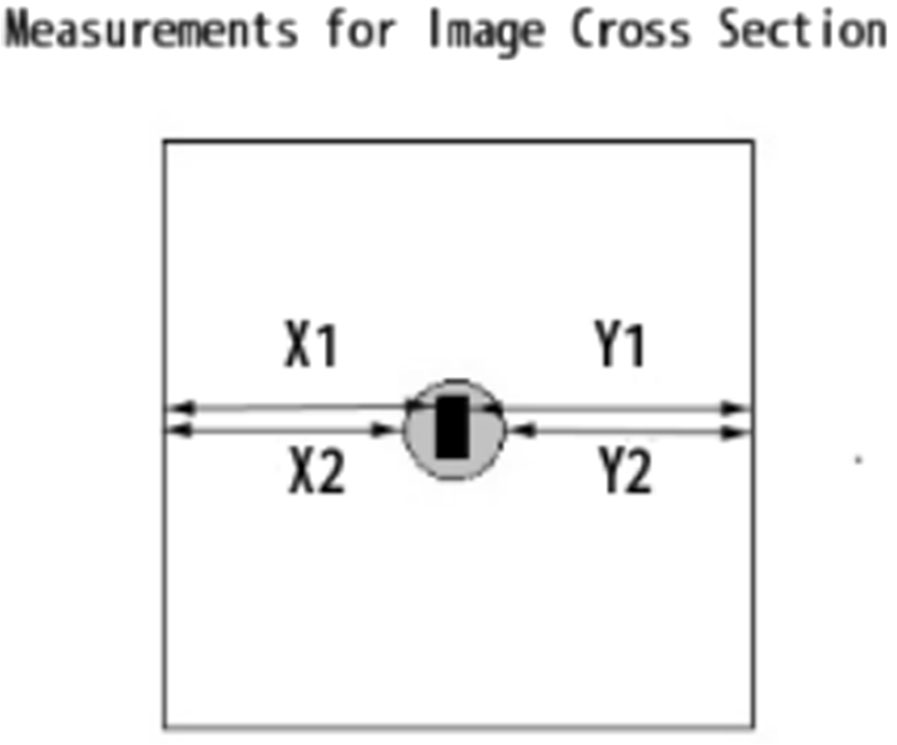
Representation drawing of tooth section showing how transportation and centring ratio were derived. Dark shaded area represents original canal space, and grey shaded area represents canal’s shape after instrumentation. X1 = shortest distance from the distal edge of the blocks to the distal periphery of the pre-instrumented canal; *X*2 = minimum distance between the distal of the blocks to the distal periphery of the instrumented canal; Y1 = shortest distance from the mesial edge of the blocks to the corresponding periphery of the pre-instrumented canal; and Y2 = the minimum distance between the mesial edge of the blocks to the mesial periphery of the instrumented canal

We designated the direction of the apical foramen as distal. X1 is the shortest distance from the distal edge of the blocks to the distal periphery of the pre-instrumented canal, and *X*2 represents the minimum distance between the distal of the block to the distal periphery of the instrumented canal. Y1 is the shortest distance from the mesial edge of the blocks to the corresponding periphery of the pre-instrumented canal, and Y2 represents the minimum distance between the mesial edges of the block to the mesial periphery of the instrumented canal (Fig. 1). A result of zero indicated no canal transportation, while other results indicated transportation towards the mesial or distal aspect of the canal.

### Measurement of centring ability

The centring ratio was calculated using the following formula [17]:

$$ \left(\mathrm{X}1\hbox{--} \mathrm{X}2\right)/\left(\mathrm{Y}1\hbox{--} \mathrm{Y}2\right)\kern0.24em \mathrm{or}\kern0.24em \left(\mathrm{Y}1\hbox{--} \mathrm{Y}2\right)/\left(\mathrm{X}1\hbox{--} \mathrm{X}2\right) $$

The denominator for the formula was the larger of the two numbers (X1–*X*2 or Y1–Y2). The closer the result was to one, the better the ability of the instrument to remain centred.

### Statistical analysis

The statistical analysis was performed with SPSS (IBM SPSS Statistics 21; SPSS Inc., Chicago, IL). The mean difference and standard deviations for different parameters of the three parts of the canal were measured for each group. The Kruskal-Wallis test was performed to analyse the data due to non-normal distribution of the values. *P* < 0.05 was recognized as the level of statistical significance.

## Results

**Fig. 2:**
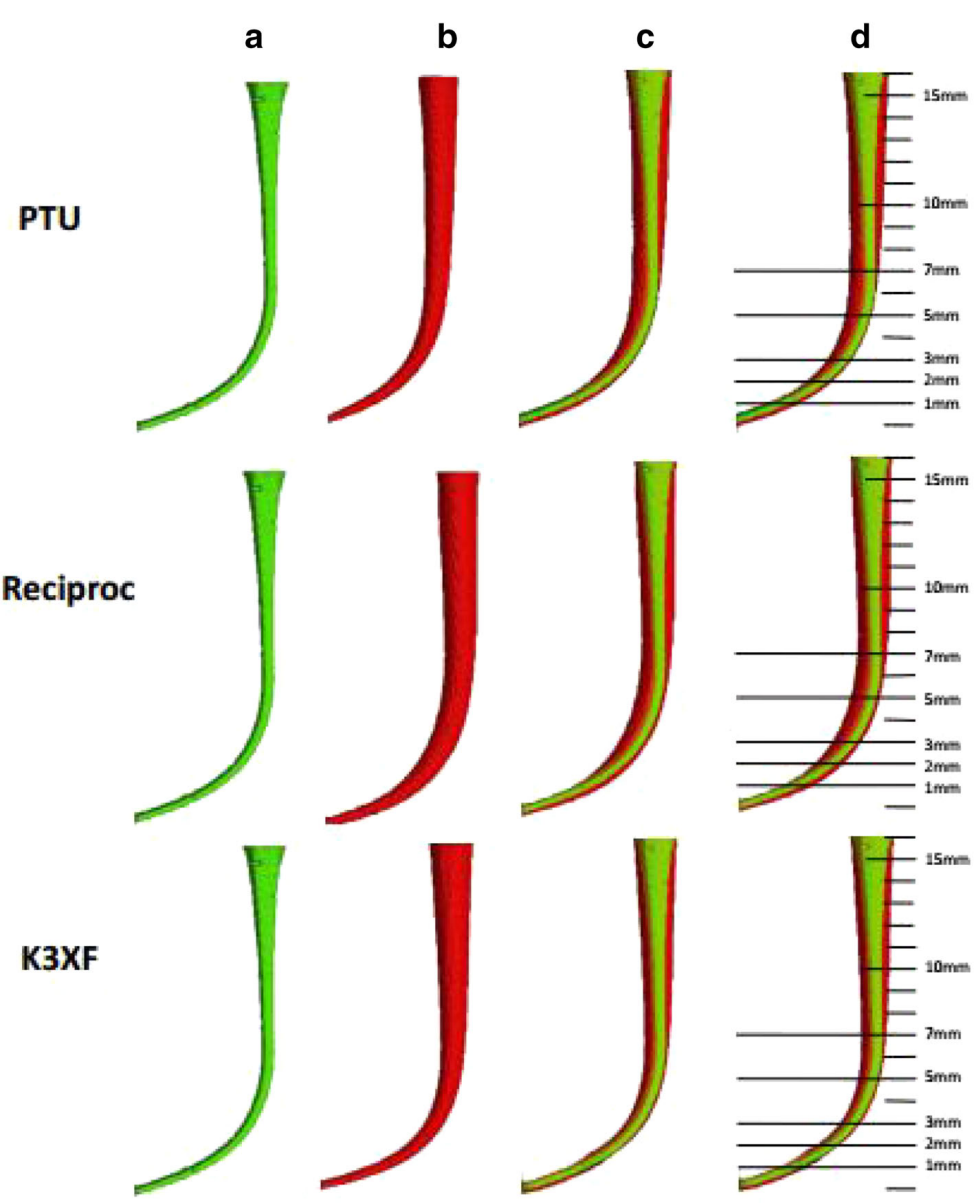
Three-dimensional panels of the simulated canals in the PTU (ProTaper Universal, top), Reciproc (middle) and K3XF (bottom) groups. **a** Preoperative reconstruction of the simulated canal; **b** Postoperative reconstruction of the simulated canal; **c** Superimposed reconstructions of the simulated canal; **d** The points at which the canal width was measured after superimposition of pre- and post-operative images. The green colour indicates the canal before instrumentation, and the red colour represents the canal after instrumentation

No instrument showed macroscopic deformation or fracture in this study. Three-dimensional moulds were reconstructed for each simulated canal (Fig. 2).

### Volume change

Table 1 shows the root volume change in the three parts of the canal before and after instrumentation. The Reciproc system produced greater volume change than the PTU and K3XF system in the apical third of the canals (*p* < 0.05). K3XF presented the smallest mean increase in apical volumetric change (*p* < 0.05).

**Table 1:** Volume (mm^3^) change in three part of the canals (mean ± SD) with different files

Group (*n* = 10)	Apical third	Middle third	Coronal third
PTU^†^	0.15 ± 0.03	0.36 ± 0.03	0.40 ± 0.07
Reciproc	0.21 ± 0.05*	0.38 ± 0.04	0.42 ± 0.05
K3XF	0.09 ± 0.01*	0.24 ± 0.04	0.32 ± 0.09

^†^, ProTaper Universal

* Mean values represented by different superscript letters are significantly different according to the Kruskal-Wallis test (*P* < .05)

### Canal transportation

The difference of transportation in different parts of the canals between PTU and Reciproc was not statistically significant, which is shown in Table 2. Additionally, significantly less transportation was noted in the K3XF group.

**Table 2:** Canal transportation (μm) of the canals after instrumentation at different measurement levels (mean ± SD)

Group (*n* = 10)	Canal transportation (mm from the apex)				
	1 mm	2 mm	3 mm	5 mm	7 mm
PTU^†^	0.03 ± 0.02	0.04 ± 0.02	0.06 ± 0.02	0.02 ± 0.02	0.03 ± 0.02
Reciproc	0.04 ± 0.02	0.06 ± 0.03	0.06 ± 0.03	0.03 ± 0.02	0.02 ± 0.02
K3XF	0.02 ± 0.01	0.01 ± 0.01*	0.02 ± 0.01*	0.01 ± 0.01	0.02 ± 0.01

^†^, ProTaper Universal

* Mean values represented with different superscript letters are significantly different according to the Kruskal-Wallis test (*P* < .05)

### Centring ratio

As shown in Table 3, the K3XF group showed significantly better centring ability than the PTU and Reciproc groups.

**Table 3:** Different measurement levels of centring ratio values for tested groups (mean ± SD)

Group (*n* = 10)	Centring ratio (mm from the apex)				
	1 mm	2 mm	3 mm	5 mm	7 mm
PTU^†^	0.76 ± 0.19	0.69 ± 0.17	0.52 ± 0.15	0.79 ± 0.17	0.72 ± 0.11
Reciproc	0.70 ± 0.12	0.54 ± 0.11	0.53 ± 0.15	0.80 ± 0.12	0.84 ± 0.11
K3XF	0.77 ± 0.15	0.82 ± 0.10*	0.81 ± 0.13*	0.86 ± 0.13	0.84 ± 0.13

^†^, ProTaper Universal

* Mean values represented with different superscript letters are significantly different according to the Kruskal-Wallis test (*P* < .05)

### Instrumentation time

The Reciproc was significantly faster than PTU and K3XF, and the time spent by the PTU was the longest one (*p* < 0.05). The mean instrumentation time was 121.6 ± 11.9 s for PTU, 74.3 ± 4.2 s for K3XF and 73.8 ± 10.3 s for Reciproc.

## Discussion

The present study describes a new method for precisely measuring canal contour before and after canal preparation in vitro. In this study, all of the simulated canals were reconstructed successfully via the radiopaque contrast technique. This method has a significant advantage over the existing methods because it allows for the non-invasive 3D evaluation of the samples with a standardization of canal length, radius and degree of curvature, taper and diameter of the apical foramen. The credibility of resin blocks for analysis of endodontic preparation and preparation techniques had been validated by previous study [9] and the materials were easily acquired, even though there were limitations with this model. A major drawback of using rotary instruments in resin blocks is the different hardness between resin and dentin. Another disadvantage is the heat generated, which might distort the canal, reduce the cutting efficiency and lead to separation of the instrument. In order to rectify this situation, syringe irrigation was applied continually during root canal preparation [18]. This method was thought to be the first attempt to compare the shaping ability of PTU, Reciproc, and K3XF by reconstructing simulated canals via the radiopaque contrast technique. This kind of model has wide application prospects in future researches. Firstly, the new method could be widely used in evaluation of root canal preparation techniques. In addition, simulated root canals included a wide range of items and C-shaped canals or lateral canals in clear resin blocks could be constructed based on the technique described by Dummer et al. [19]. Therefore, it should be possible to evaluate the quality of gutta-percha filling, the irrigating efficiency in standardized complex root canals. Moreover,it also has advantages in pre-clinical dental education because of easiness of sample obtainment.

The results of the present study revealed that the use of Reciproc instruments resulted in significantly greater volumetric change in the apical part of root canals. Reciproc R25 has the same tip taper and diameter as PTU F2 at 3 mm from their working tips. You et al. evaluated the shaping ability of continuous rotating motion in comparison to reciprocating motion in curved canals and the results showed that the type of motion had little effect on changes in root canal volume and curvature [20]. Thus, different modes of motion may not explain our study’s results, but differences in instrument designs on cross-section and cutting angles may lead to this result. Reciproc system has a double-cutting edge with S-shape geometry. Previous studies have confirmed that instruments with S-shape cross sections were associated with enhanced cutting efficiency [21, 22]. Another important attribute of instruments is debris removal to release the clogged cutting blades. S-shaped cross-sections allow for more space between the canal walls and the instrument [23]. The lowest volume change was recorded when K3XF instruments were used. This may be partially explained by the smallest apical taper of K3XF (size 25/0.06).

The enlargement of the apical area is conducive to reducing or eliminating the number of microorganisms in complex root canals. Gomes et al. concluded that the mechanical action of instruments eliminated more than 47% of oral bacterial endotoxins against the dentin wall [24]. On the one hand, with more volumetric changes, the irrigating needle can be placed into deeper apical sites, yielding a better irrigating effect [25, 26]. It is widely accepted that irrigation with the flow and backflow of the flushing fluid is directly related to the disinfection effect of root canals [27]. Nevertheless, previous studies noted that dentin thickness could affect the incidence of vertical root fracture, and it is safe for dentists when the canal width was controlled within 30% of the total root width [28].

Canal transportation and centring ability could be influenced by factors including different designs of instruments and the type of alloy [29, 30]. K3XF exhibited the least transportation and the best centring ability at 2 and 3 mm from the apical foramen in this study. This may be explained by smallest apical taper of K3XF (size 25/.06) compared with PTU F2 and Reciproc R25 (size 25/.08). Furthermore, R-phase (K3XF) technology makes the alloy more flexible than M-wire (Reciproc) and conventional alloy (PTU), which can minimize canal transportation in curved canals [29]. Wu et al. reported that apical transportation over 0.3 mm could compromise the outcome of the root canal filling [31]. In our study, all the transportation values obtained from different instruments were not over 0.3 mm. There were no significant differences in canal transportation and centring ability between the Reciproc and PTU groups, which were coincident with studies conducted by Capar et al. and Bürklein et al. [32, 33]. One of the reasons is that both of the instruments have non-cutting tips that only act as a guide to penetration [34]. However, Yoo et al. found that the Reciproc R25 maintained the original canal curvature better than the PTU system up to F2 in standardized plastic blocks [35]. This can be explained by the different study models used.

Instrumentation time relies on the numbers of used files, the technique and the experience of the operator. In our study, the numbers of the used files was the most influential factor. Obviously, Reciproc, which is a single-file system, required less time for instrumentation than PTU and K3XF that may because of the omission of the file change. This result was in agreement with previous studies [32, 33, 35].

## Conclusions

The combination of μCT and simulated canals provided a method for the evaluation of the shaping properties of NiTi rotary instruments. Under the conditions of our study, the null hypothesis was rejected. PTU, Reciproc and K3XF instruments shaped simulated canals without any significant shaping error. Reciproc resulted in increased apical volume changes and significantly shorter instrumentation time compared with K3XF and PTU. K3XF exhibited less transportation and better centring ability at 2 and 3 mm from the apical foramen than PTU and Reciproc.

## Additional file

Additional file 1: Original data. Relevant data underlying the findings described in manuscript. (XLS 25 kb)

## Abbreviations

NiTi: Nickel-titanium
PTU: ProTaper Universal
μCT: Micro-CT
